# Femtosecond Laser-Induced Periodic Surface Structures in Titanium-Doped Diamond-like Nanocomposite Films: Effects of the Beam Polarization Rotation

**DOI:** 10.3390/ma16020795

**Published:** 2023-01-13

**Authors:** Sergei M. Pimenov, Evgeny V. Zavedeev, Beat Jaeggi, Beat Neuenschwander

**Affiliations:** 1 Prokhorov General Physics Institute of the Russian Academy of Sciences, 119991 Moscow, Russia; 2 Institute for Applied Laser, Photonics and Surface Technologies ALPS, Bern University of Applied Sciences, CH-3400 Burgdorf, Switzerland

**Keywords:** Ti-DLN films, femtosecond laser ablation, linear polarization, laser-induced periodic surface structures (LIPSS), lateral force microscopy

## Abstract

We study the properties of laser-induced periodic surface structures (LIPSS) formed on titanium-doped diamond-like nanocomposite (DLN) a-C:H:Si:O films during ablation processing with linearly-polarized beams of a visible femtosecond laser (wavelength 515 nm, pulse duration 320 fs, pulse repetition rates 100 kHz-2 MHz, scanning beam velocity 0.05–1 m/s). The studies are focused on (i) laser ablation characteristics of Ti-DLN films at different pulse frequencies and constant fluence close to the ablation threshold, (ii) effects of the polarization angle rotation on the properties of low spatial frequency LIPSS (LSFL), and (iii) nanofriction properties of the ‘rotating’ LIPSS using atomic force microscopy (AFM) in a lateral force mode. It is found that (i) all LSFL are oriented perpendicular to the beam polarization direction, so being rotated with the beam polarization, and (ii) LSFL periods are gradually changed from 360 ± 5 nm for ripples parallel to the beam scanning direction to 420 ± 10 nm for ripples formed perpendicular to the beam scanning. The obtained results are discussed in the frame of the surface plasmon polaritons model of the LIPSS formation. Also, the findings of the nanoscale friction behavior, dependent on the LIPSS orientation relative to the AFM tip scanning direction, are presented and discussed.

## 1. Introduction

The pioneering studies of diamond-like nanocomposite (DLN) a-C:H:Si:O films [1,2,3] had evidenced the amorphous DLN structure to be an ideal dielectric matrix for introducing different metals (such as W, Ti, Cr, Mo, Hf, Nb, Ta) during plasma-assisted chemical vapor deposition from siloxane precursors. The doping with metals provided unique structural and electronic properties (e.g., variation of the electrical conductivity over 18 orders of magnitude, from ~10^14^ to 10^−4^ Ω⋅cm), and exceptional thermal and chemical stability of the electrically conductive DLN films [2,3,4,5,6,7,8,9]. High-resolution transmission electron microscopy examination of metal-doped DLN films [9,10,11] revealed the formation of metal carbide nanocrystals and the increase of the nanocrystal size with the metal concentration in the films. Both the high thermal/chemical stability and formation of the metal carbide nanocrystals in the dielectric matrix proved to be important factors in laser surface structuring experiments. The first demonstration of laser surface structuring of highly doped W-DLN films with a micron spatial resolution was related to selective-area diamond deposition onto a laser-micropatterned W-DLN film at the high temperature (~800 °C) and harsh environment of the methane-hydrogen plasma [12]. Interestingly, this was an example of an ‘indirect’ structuring, as the diamond microstructures were grown on unirradiated surface areas (seeded with diamond nanoparticles) due to the high chemical stability of the W-DLN film. In the direct laser processing and microstructuring of W-DLN films, the presence of the metal carbide phase and its content in the films was reported to noticeably influence the ablation characteristics, especially at the infrared (IR) laser wavelength [13]. It is certain to be a consequence of the composite structure properties characterized by much higher optical absorption of the metal carbide nanoparticles than of the dielectric matrix.

Recently, the key role of the composite structure was clearly pronounced in femtosecond (fs) IR laser processing/nanostructuring of titanium-doped DLN films, surface graphitization and formation of laser-induced periodic structures (LIPSS) at sub-threshold fluences [14], as compared to the fs-LIPSS formed in DLN films [15]. The LIPSS is known as an extraordinary laser-physics phenomenon and an advanced technology of the surface nanostructuring and functionalization of different materials [16,17,18,19]. The studies of fs-laser nanostructuring and LIPSS formation on DLN films followed the works on fs-laser microstructuring of the films, which showed the fs-laser ablation processing very efficient to control friction at the micro and macroscale [20,21,22], wetting properties [23], and to improve tribological properties of laser-microstructured films in lubricated sliding [24,25]. For metal-doped DLN films, there were no systematic, application-oriented investigations of fs-laser ablation processing and microstructuring, except for an interesting finding of the anisotropic wetting behavior of a microgrooved Ti-DLN surface [23]. Of interest is also a question of using high pulse frequencies during fs-laser processing of Ti-DLN films, briefly discussed in [14] and required for high throughput in many micromachining applications. The studies of the fs-laser nanostructuring and LIPSS formation on DLN and Ti-DLN films [14,15], and on other types of diamond-like carbon (DLC) films [26,27,28,29], were mainly concerned with the mechanism of the LIPSS formation, with only a few papers related to tribological applications of the LIPSS-structured films [30,31,32].

In this paper, we study the formation of LIPSS on Ti-DLN films during ablation processing with linearly-polarized beams of a visible fs-laser (wavelength 515 nm, pulse duration 320 fs, pulse repetition rates 100 kHz–2 MHz, scanning beam velocity 0.05–1 m/s) as a function of the linear polarization direction. The direction of the beam polarization ***E*** relative to the beam scanning direction ***V_s_*** is varied from ***E*** ⊥ ***V_s_*** to ***E*** ‖ ***V_s_*** with a ‘rotation’ step of 30° (***E*** is the electric field, ***V_s_*** is the scanning beam velocity). The studies are focused on (i) laser ablation characteristics of Ti-DLN films at different pulse frequencies and constant fluence exceeding the single-pulse ablation threshold, (ii) effects of the beam polarization rotation on the properties of low spatial frequency LIPSS (LSFL) formed on the Ti-DLN films at the pulse frequencies of 500 kHz and 100 kHz, and (iii) nanofriction properties of the ‘rotating’ LIPSS using atomic force microscopy (AFM) in a lateral force mode. New findings of the LSFL properties changed with the polarization angle and of the nanoscale friction behavior, dependent on the LIPSS orientation relative to the AFM tip scanning direction, are presented and discussed.

## 2. Materials and Methods

### 2.1. Structure and Properties of Ti-DLN Films

**Figure 1:**
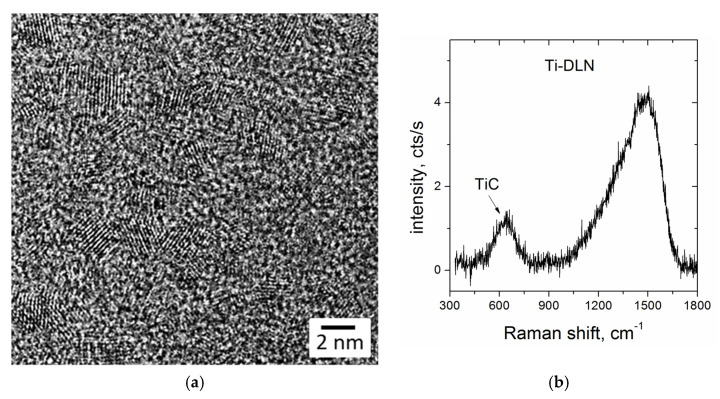
HRTEM image (**a**) and Raman spectrum (**b**) of the Ti-DLN films (with the titanium concentration of 17–18 at.%).

DLN films were grown on Si substrates using a technique of plasma-assisted chemical vapor deposition (PACVD) from polymethylphenylsiloxane (PMPS) vapors. The characteristics of the CVD system were described elsewhere [11]. Briefly, the PACVD setup had two different sources (plasmatron and magnetron) for precursor supply into a vacuum chamber that allowed both the undoped and metal-doped DLN films to be deposited onto various substrates. The high-frequency potential (1.76 MHz, 0.1–1.5 kV) applied to the substrate holder provided the appearance of a negative constant bias voltage on the substrate; the bias voltage was in the range of −200 to −700 V. Metal-containing DLN films were produced by simultaneous deposition of a carbon-silicon matrix from PMPS vapor plasma and magnetron sputtering of a titanium target in an argon atmosphere. The film growth rate was 1–2 µm/h. Si(100) plates of 20 mm × 20 mm × 0.52 mm size were used as the substrates. The Ti-DLN films of 3–5 µm thickness, titanium content of 17–18 at.% and nanoindentation hardness of 22–23 GPa were used in the laser structuring experiments. The structure of the Ti-DLN films is characterized by the formation of TiC nanocrystals in the amorphous DLN matrix, proved by high-resolution transmission electron microscopy (HRTEM) and Raman spectroscopy analysis of the films [11]. Figure 1a shows an HRTEM image of a cross-sectional region with TiC nanocrystals of 2–3 nm size, typical of the Ti-DLN films with high Ti contents of 15–18 at.%. A Raman spectrum of the Ti-DLN film (shown in Figure 1b) contains a broad low-intensity peak at ~600 cm^−1^ attributed to the TiC phase [33]. Specifics of the Raman spectra measurements in highly-doped Ti-DLN films caused by strongly decreased scattering intensity are discussed in more detail in ref. [11]. The presence of TiC nanocrystals influences the optical properties of Ti-DLN films due to the difference in the light absorption coefficients (*α*) of the DLN matrix and TiC crystallites, equal to the *α_DLN_* = (3.3 − 5.6) × 10^4^ cm^−1^ [14] and *α_TiC_* = 5.9 × 10^5^ cm^−1^ [34] at the laser wavelength λ = 515 nm.

### 2.2. Femtosecond Laser Processing of Ti-DLN Films

**Figure 2:**
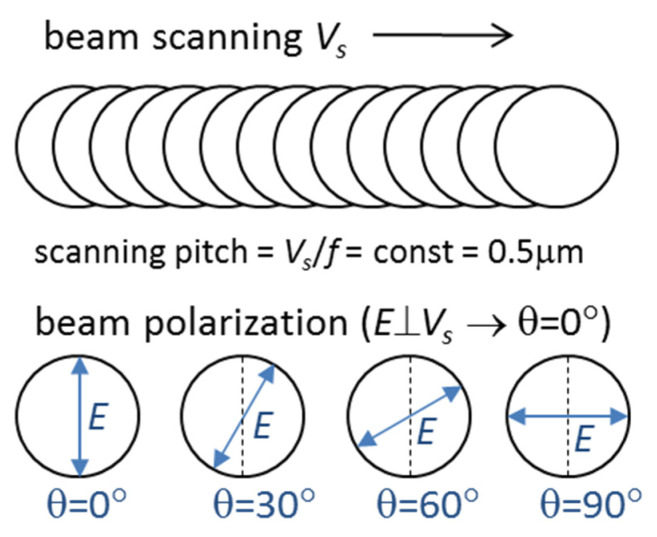
Scheme of laser experiments with rotation of the beam polarization relative to the beam scanning direction (*V_s_* is the scanning velocity and *E* is the electric field of the linearly-polarized laser beam).

Laser processing of the films was carried out using a SATSUMA HP2 femtosecond laser system [35,36] (from Amplitude Systèmes, Pessac, France) generating pulses of τ = 320 fs duration at the wavelength λ = 515 nm. The average power (*P*) was varied from 25 to 500 mW at the pulse repetition rate (*f*) changed from 100 kHz to 2 MHz, with the pulse energy (*ε_p_*) of 0.25 μJ being constant. The laser beam was focused with a 100-mm telecentric objective, and the beam radius was *w_o_* = 7.1 µm at the 1/e^2^ level. The peak fluence *F* = 2*ε_p_*/*π*$w_{0}^{2}$ amounted to *F* = 0.32 J/cm^2^, being slightly higher than the single-pulse ablation threshold *F_th_* = 0.3 J/cm^2^ in DLN films [21] and also in Ti-DLN films, as the ablation threshold was reported to decrease with metal doping [13]. A high-precision galvanometer scanner intelli*SCAN*_se_ from Scanlab (Puchheim, Germany) was applied to control the scanning beam velocities (*V_s_*) and to produce microgrooves of ≈10 μm width and 15 mm length, with the LIPSS formed on the groove surface at different irradiation conditions. At the *f* = 100 kHz, the scanning velocity was *V_s_* = 0.05 m/s and the pitch distance amounted to *V_s_/f* = 0.5 µm. At higher pulse repetition rates of 500 kHz, 1 MHz and 2 MHz, the scanning velocity was proportionally increased to 0.25, 0.5 and 1 m/s, in order to keep the values of the pitch distance *V_s_/f* = 0.5 µm and effective pulse number *N_eff_* = 2*w_o_*/(*V_s_/f*) in the study of the effects of pulse frequency/heat accumulation on the ablation rates and properties of laser-nanostructured surfaces obtained at the *F* = 0.32 J/cm^2^. All microgrooves were produced by 2 scans of the laser beam along each groove. The laser beam was linearly polarized, and the direction of the beam polarization ***E*** relative to the beam scanning direction ***V_s_*** was varied from ***E*** ⊥ ***V_s_*** to ***E*** ‖ ***V_s_*** with a ‘rotation’ step of 30° (***E*** is the electric field vector, ***V_s_*** is the scanning velocity vector). The polarization angle (θ) was taken equal to θ = 0° for ***E*** ⊥ ***V_s_***, then it was changed to θ = 30°, θ = 60° and finally to θ = 90° for ***E*** ‖ ***V_s_***. A scheme of the laser structuring experiments with rotation of the beam polarization is shown in Figure 2. All experiments were carried out in ambient air at the normal beam incidence onto the film surface.

The surface morphology and dimensions of laser-produced microgrooves on the Ti-DLN films were examined using laser scanning microscopy (LSM), scanning electron microscopy (SEM) and atomic force microscopy (AFM). The data of laser-induced structure transformation in the Ti-DLN surface was obtained from Raman spectra measured using a LabRAM HR Evolution spectrometer (Horiba, Japan) at the excitation wavelength 532 nm. The two-dimensional Fast Fourier Transform (FFT) analysis of SEM and AFM images was applied to determine the periods of the LIPSS formed.

**Figure 3:**
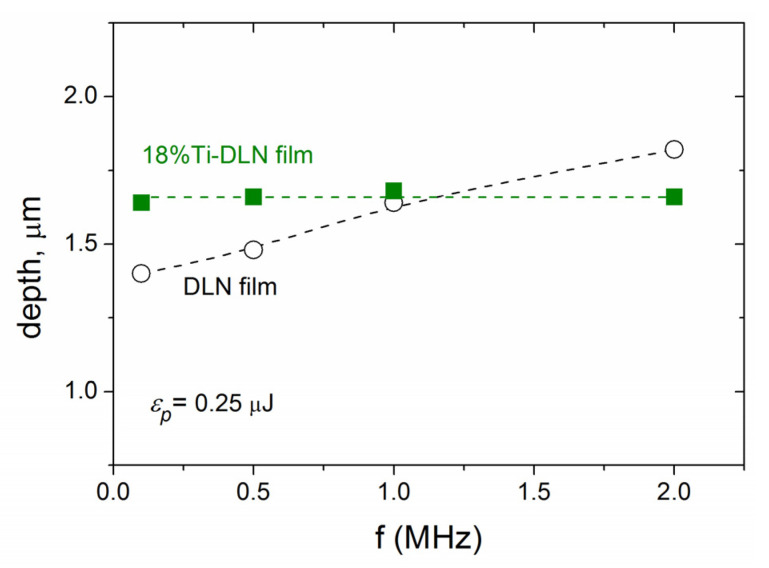
Influence of the pulse frequency on the ablation depth of microgrooves formed on the Ti-DLN film at the identical parameters of the pulse energy *ε_p_* = 0.25 μJ and effective pulse number *N_eff_* = 2*w_o_*/(*V_s_/f*), compared to the ablation depth vs. pulse frequency for the DLN film (***E*** ‖ ***V_s_***).

**Figure 4:**
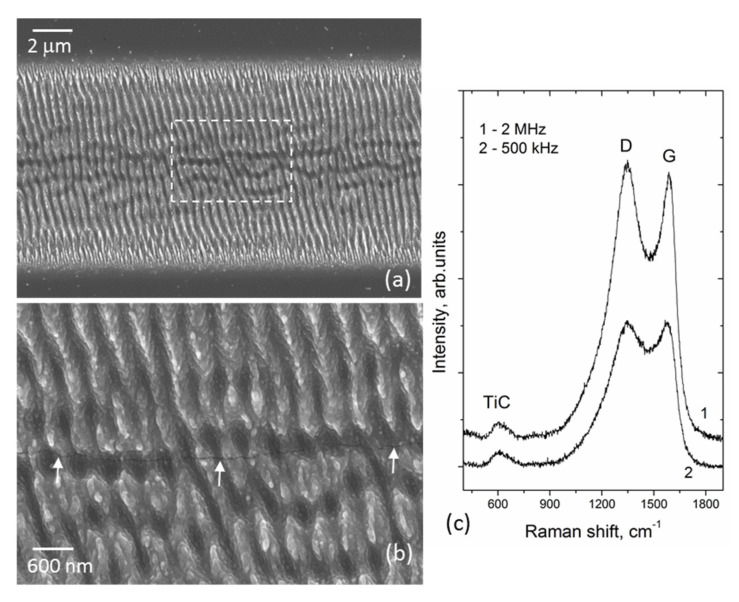
(**a**) SEM image of a microgroove formed on the 18%Ti-DLN film at *f* = 2 MHz, *ε_p_* = 0.25 μJ and *V_s_* = 1 m/s (***E*** ‖ ***V_s_***); (**b**) SEM image of a marked area in the groove center, with the arrows indicating the crack formation along the groove bottom; (**c**) Raman spectra of the microgrooves formed at *f* = 2 MHz and *f* = 500 kHz.

An increase of the pulse frequency (and average power) in high quality laser microprocessing of various materials with ultrashort pulses is caused by the need of high throughput of the laser technologies, especially in the field of industrial applications [35]. For amorphous carbon coatings there are limitations of using high pulse frequencies, dealing with the surface quality in/around laser-produced microstructures due to enhanced graphitization and redeposition of ablated nanoparticles and spallation fragments [22,23]. It is therefore needed to determine the optimum high-pulse-frequency regimes, which improve the laser processing characteristics and keep the acceptable surface quality of the micro/nanostructures. The data, demonstrating the positive and negative effects of using the MHz pulse frequencies for fs-LIPSS fabrication on highly-doped Ti-DLN film, are presented in Figure 3 and Figure 4.

Figure 3 shows the ablation depth vs. pulse frequency dependence for the Ti-DLN films in comparison with that for a metal-free DLN film. It is seen that the ablation depth is practically constant (≈1.65 μm) in the range of *f* = 0.1–2 MHz, presumably due to a higher thermal diffusivity of the metal-doped film. So the beneficial effect of the higher pulse frequencies is only in the shortened processing time, not in the increased ablation rate. The main negative effect is the crack formation along the groove bottom during ablation and LIPSS formation at *f* = 2 MHz, shown in Figure 4a,b. In addition, the enhanced surface graphitization of the Ti-DLN film follows from the comparison of Raman spectra (in Figure 4c) of the grooves formed at *f* = 2 MHz and *f* = 500 kHz. The Raman spectra of the grooves formed at the lower pulse frequencies (*f* = 100 kHz and *f* = 500 kHz) were reported [14] to be characterized by three peaks: D-peak at 1350 cm^−1^, G-peak at 1580 cm^−1^, and TiC peak at ~600 cm^−1^. The higher intensity ratio of the D and G peaks together with a shift of the G peak to the higher frequency (~1590 cm^−1^) evidence the enhanced surface graphitization in the groove formed at *f* = 2 MHz. The similarity of the TiC peaks is likely to evidence that the content of the TiC phase in the surface layer is not changed during ablation at different pulse frequencies. Both the destruction of the coating along the groove center and higher graphitization degree result from higher surface temperatures at *f* = 2 MHz. For the above reasons, laser experiments on the LIPSS formation at different angles of the beam polarization were carried out at the lower pulse frequencies *f* = 100 kHz and *f* = 500 kHz.

### 2.3. Lateral Force Microscopy of Laser-Structured Ti-DLN Films

The surface relief and nanoscale friction properties of the laser-structured Ti-DLN film with the ‘rotating’ LIPSS were studied with an atomic force microscope of the NTEGRA Spectra system (NT-MDT, Moscow, Russia) using the contact-mode AFM techniques—lateral force microscopy (LFM) and force-distance curve measurements. The LFM technique allows the surface relief and lateral (friction) force to be measured simultaneously during tip scanning [37,38] that enables us to compare the friction forces in laser-structured and original surface areas of the films. Measurements of the force-distance curves [39] are used to determine the capillary forces between the AFM tip and film surface, which add an extra load on the tip and can remarkably enlarge the friction force [21,40]. Diamond-coated Si probes with a spring constant of 1.4 N/m and tip radius of *R_tip_*~100 nm were used in order to reduce the tip wear during scanning and capillary forces [40]. The tip scanning was performed across the microgrooves (containing the LIPSS of different orientations) at the tip load of 200 nN and scanning velocity of 15 µm/s. The LFM measurements were carried out in ambient air at relative humidity RH = 62% and room temperature T = 25 °C. In addition to the LFM examination, AFM images of the LIPSS with a higher resolution were recorded in a tapping mode using sharp silicon probes with a tip radius < 10 nm

## 3. Results and Discussion

### 3.1. Influence of the Beam Polarization Rotation on the LIPSS Properties

**Figure 5:**
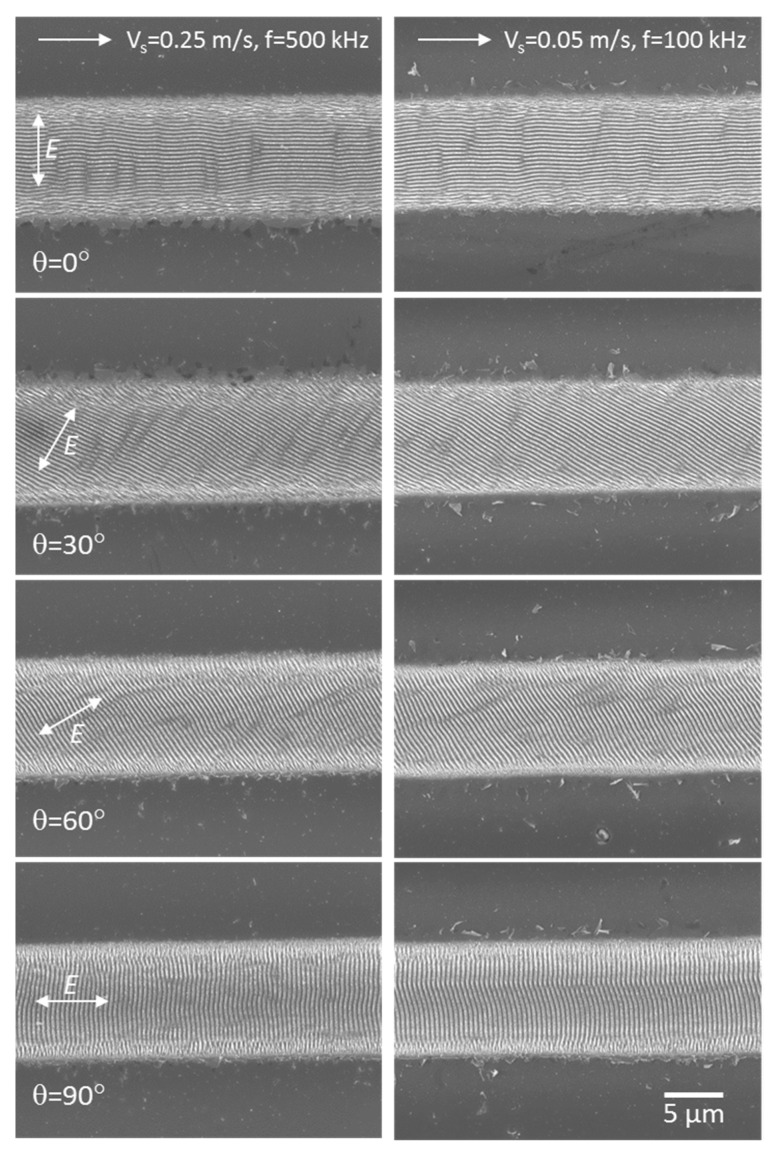
SEM images of the microgrooves formed on the 18%Ti-DLN film (3.6 μm thick) by fs-laser processing at *F* = 0.32 J/cm^2^, pulse frequencies *f* = 500 kHz (**left images**) and *f* = 100 kHz (**right images**), and different angles of the beam polarization.

The general view of microgrooves formed at two pulse frequencies (*f* = 500 kHz and *f* = 100 kHz) and different angles of the beam polarization is shown in SEM images in Figure 5. It is seen in the images that the rotation of the electric field vector (from θ = 0°, ***E*** ⊥ ***V_s_***) with a step of 30° relative to the beam scanning direction leads to the ‘synchronous’ rotation of the LIPSS on the film surface. The microgrooves fabricated at the two pulse frequencies are pronounced to be very similar, with a somewhat larger amount of ablated particles/spallation fragments around the grooves formed at the *f* = 100 kHz. So the factors of the processing time and surface quality around the microstructures were the reasons for more detailed investigations of the LIPSS produced at *f* = 500 kHz, including contact and noncontact modes of AFM.

**Figure 6:**
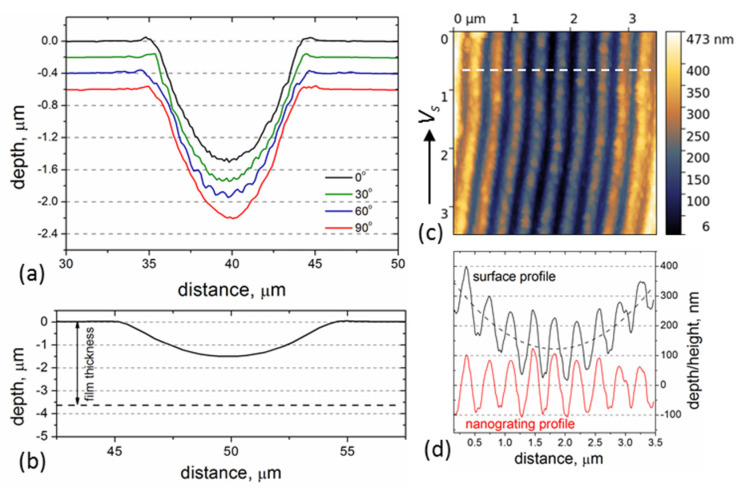
(**a**) AFM surface profiles across the microgrooves produced at different polarization angles and *f* = 500 kHz. (**b**) The true groove profile on the film surface, i.e., the scale along X and Y axes is the same. (**c**) AFM image of the LIPSS measured in a tapping mode with a sharp Si tip (***E*** ⊥ ***V_s_***). (**d**) The surface profile along the dashed line in (**c**), nanograting profile is obtained by subtraction of the fitted curve (dashed line) from the surface profile.

The rotation of the beam polarization had a minor influence on the ablation depth/rate, as is seen in the AFM surface profiles (in Figure 6a) measured across the microgrooves using a diamond-coated tip in the contact-mode. The ablation depth was found to slightly change with the polarization angle in the range of 1.5–1.6 µm. Figure 6b shows the true groove profile just to underline that (i) the fs-LIPSS are produced at a quite large distance from the film/substrate interface, and (ii) the slope of the groove surfaces is changed from the center to the edges (by ~30°). The data of the nanograting depth is obtained from AFM images of the LIPSS recorded with sharp Si tips in the central areas of the grooves. As an example, the AFM data are presented in Figure 6c,d for the beam polarization ***E*** ⊥ ***V_s_***. The nanograting profile becomes more clearly pronounced after subtraction of a polynomial (quadratic) background from the surface profile, as shown in Figure 6d. The LIPSS depth is then determined to be of 140–210 nm. For the other LIPSS orientations, the grating depth values are somewhat smaller, being in the range of 130–180 nm.

**Figure 7:**
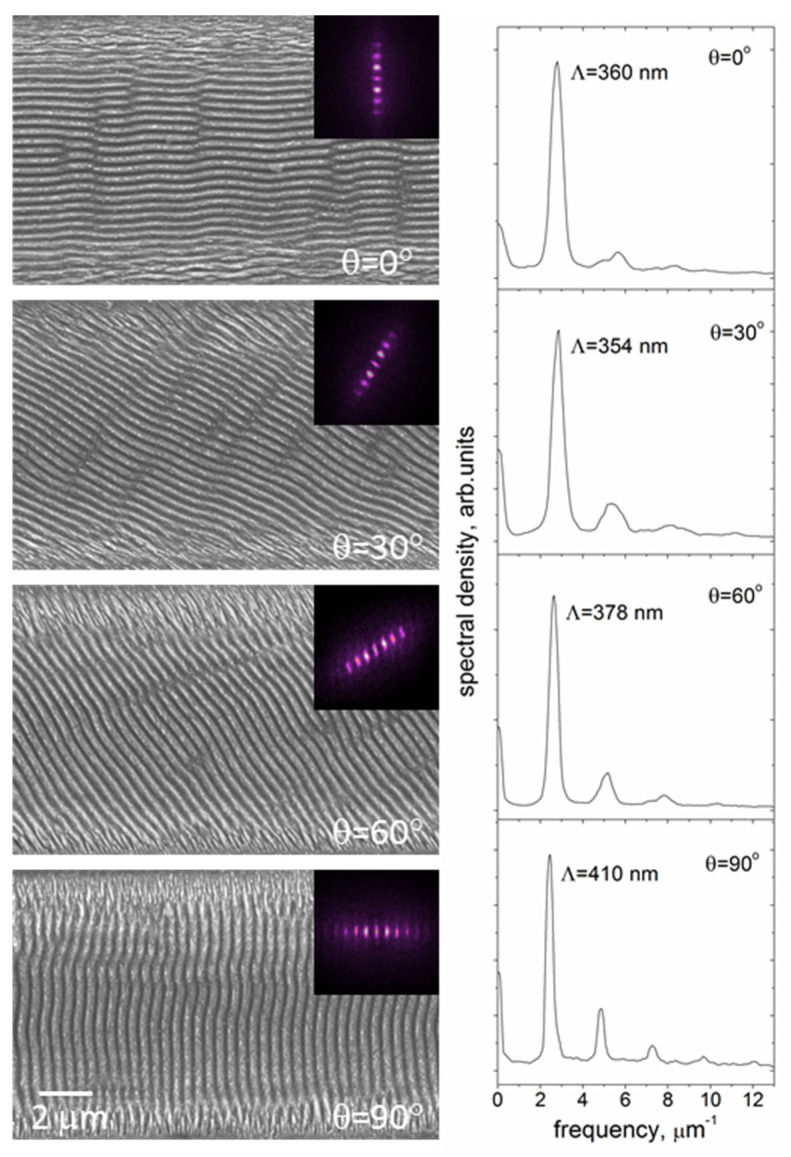
SEM images and FFT patterns/spectra of the LIPSS formed on the 18%Ti-DLN film by fs-laser processing at *F* = 0.32 J/cm^2^, *f* = 500 kHz, *V_s_* = 0.25 m/s and different angles of the beam polarization. The size of the FFT patterns is 30 µm^−1^ × 30 µm^−1^. The beam scanning direction is along the X axis direction.

The results of the FFT analysis of SEM images of the microgrooves produced at different polarization angles are presented in Figure 7. It is seen in the FFT patterns that the grating vector of the LIPSS formed at different polarizations is rotated with the electric field vector, with the ripples being oriented normally to the electric field of the linearly-polarized laser beam. The FFT spectra indicate an increase of the LIPSS period with the polarization angle. Note, the appearance of higher harmonics (quasi-periods Λ/2, Λ/3, Λ/4, Λ/5) in the FFT spectra becomes more discernible with the beam polarization rotation from ***E*** ⊥ ***V_s_*** to ***E*** ‖ ***V_s_***, which evidence that the shape of nanoripples is becoming more different from a sinusoidal one; similar tendencies were observed in the FFT spectra of AFM images.

**Figure 8:**
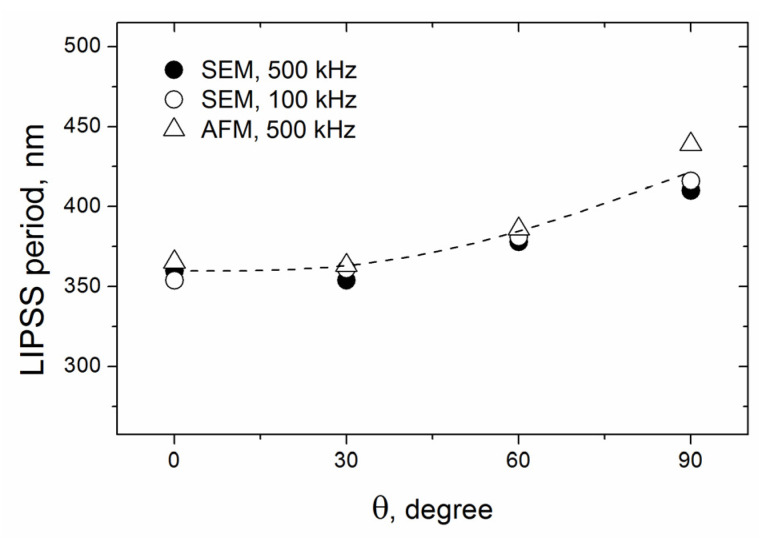
LIPSS period vs. angle of the beam polarization for two pulse frequencies; the LIPSS periods were obtained from the FFT spectra of the SEM and AFM images.

The data of the LIPSS periods vs. polarization angle are summarized in Figure 8 for the two pulse frequencies *f* = 500 kHz and *f* = 100 kHz. First, it is seen that the periods are practically the same for the two frequencies. Second, the LIPSS periods of Λ = 360 ± 5 nm are nearly constant in the range of the polarization angles θ = 0–30°, and then the periods are gradually increased to the values of Λ = 380 ± 2 nm at θ = 60° and Λ = 420 ± 10 nm for the ripples formed perpendicular to the beam scanning direction (θ = 90°). The Λ values are larger than λ/2, corresponding to the formation of low spatial frequency LIPSS (LSFL) (the term used in conventional LIPSS classification [16,17]) in the Ti-DLN films at the condition of *F > F_th_*.

The LSFL formation is usually considered in the frame of the model of surface plasmon polaritons (SPP) excitation at the air/metal interface [17,41]. The Ti-DLN film is a nanocomposite material consisting of TiC nanocrystals in the dielectric DLN matrix transformed in the surface layer into the conductive GC matrix during fs-laser processing, as discussed above and in ref. [14]. We consider the SPP excitation at both the air/GC interface and air/TiC interface (to see a difference between the two materials), and calculate the SPP wavenumbers using the equation [42]

(1) $$\beta =k_{0}\sqrt{\frac{\varepsilon_{1}\varepsilon_{2}}{\varepsilon_{1}+\varepsilon_{2}}}$$

where *β* is the SPP wave number, *k*_0_ is the wave vector of light in vacuum, *ε*_1_ is a dielectric function of the laser-exited material (GC or TiC) and *ε*_2_ = 1 is the dielectric constant of air.

Due to laser-excited electron-hole plasma in the surface layer, the dielectric function *ε*_1_(*ω*) is described by the Drude model [43]

*ε*_1_(
*ω*) =
*ε*_1_ −
*ω_p_*^2^/(*ω*^2^[1 + *i*/(*ωτ*)]), (2)

where *ε*_1_ is the dielectric permittivity of the unexcited material (*ε_GC_* = 2.68 + *i*2.47 [44] and *ε_TiC_* = 2.91 + *i*14.38 [34]), *ω* is the laser frequency, *τ* is the Drude damping time, *ω_p_* = [*N_e_ · e*^2^/(*ε*_0_ · m* · *m_e_*)]^1/2^ is the plasma frequency, with the free-carrier density *N_e_*, electron charge *e*, dielectric permittivity of vacuum *ε*_0_, electron mass *m_e_*, and optical effective mass of electrons m* = 0.5.

**Figure 9:**
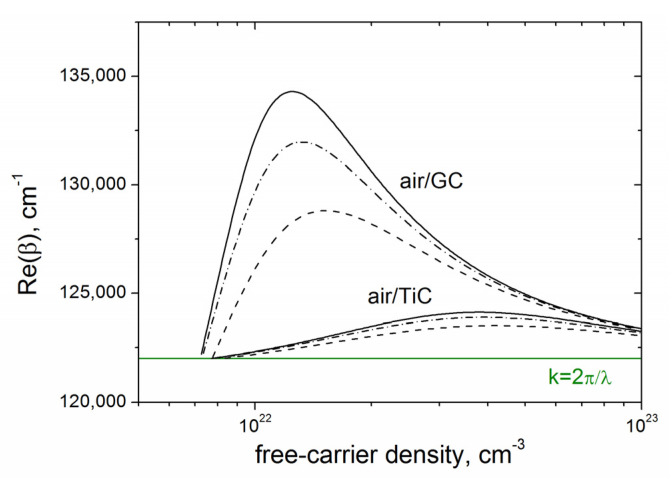
Re(β) vs. *N_e_* for the air/GC and air/TiC interfaces, λ = 515 nm: solid curves—No damping of free electrons, dashed curves—The damping time τ = 1 fs, dashed-dot curves—τ = 3 fs.

**Figure 10:**
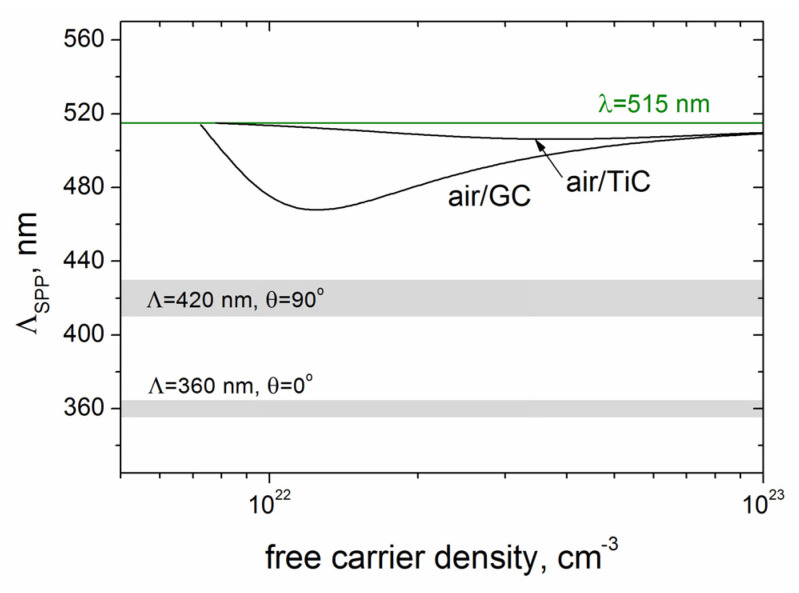
Λ_SPP_ vs. *N_e_* for the air/GC and air/TiC interfaces (the damping of free electrons is ignored); the marked grey regions correspond to the experimentally obtained LIPSS periods increased from the Λ = 360 ± 5 nm (for ***E*** ⊥ ***V_s_***) to the Λ = 420 ± 10 nm (for ***E*** ‖ ***V_s_***).

As the data of the Drude damping time in GC is lacking in the literature [28], we calculated the SPP wavenumbers for τ = 1 fs, τ = 3 fs, and τ = ∞ (i.e., the damping of free carriers was ignored). The dependences of the Re(β) vs. *N_e_* for the air/GC and air/TiC interfaces are shown in Figure 9. Even within the uncertainty in the Drude damping time, the excitation of SPPs with larger wavenumbers and smaller periods is characteristic of the air/GC interface. The SPP periods Λ_SPP_, calculated as Λ_SPP_ = 2π/Re(*β*), are compared in Figure 10 with the experimentally obtained LIPSS periods. It is seen that the smallest periods of SPP (Λ_SPP_~470 nm) to be excited at the air/GC boundary are larger than the LIPSS periods of Λ = 360–420 nm. As mentioned above and shown in Figure 6b, the slope of the groove surfaces is changed by ~30° during multipulse irradiation (the effective pulse number *N_eff_* = 2*w_o_*/(*V_s_/f*) = 28), making the angle of the laser beam incidence Θ different from the normal incidence Θ = 0° at the beginning of laser processing. So the obtained values of Λ ≈ (0.7–0.8)λ can be explained by the nonnormal beam incidence with different light polarizations and dielectric permittivity changes of the laser-excited nanocomposite layer, based on the mechanisms governing the formation and properties of fs-LIPSS considered in detail in various review papers on fs-LIPSS (see [17,19,45] and refs. therein).

### 3.2. Nanofriction Properties of the ‘Rotating’ LIPSS on the Ti-DLN Films

**Figure 11:**
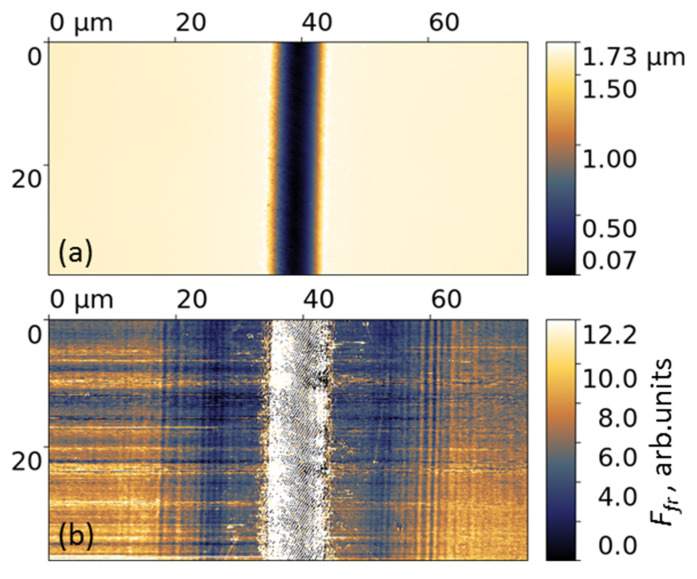
Contact-mode AFM images of the surface relief (**a**) and friction force (**b**) of the microgroove produced on the Ti-DLN film at *F* = 0.32 J/cm^2^, *f* = 500 kHz, *V_s_* = 0.25 m/s and the polarization angle θ = 30°. The diamond-coated tip is scanned across the groove (from left to right and backward) at the load 200 nN and velocity 15 μm/s in ambient air at RH = 62%.

The microgrooves formed on the Ti-DLN film at *f* = 500 kHz and different angles of the beam polarizations (shown in Figure 5 and Figure 7) were studied using lateral force microscopy, aimed to clarify a question of how the periodical relief and its orientation would influence the nanoscale friction behavior. The typical images of the surface relief and friction force are shown in Figure 11 for the microgroove with the LIPSS rotated by θ = 30° (see Figure 7). The tip scanning area (X × Y) was 75 µm × 37.5 µm in the LFM tests. It is clear that because of the large scanning area and groove depth (of 1.5–1.6 μm) the surface grating is not ‘visible’ on the relief image in Figure 11a, as compared to the AFM image in Figure 6c. The diamond-coated Si tips were specially used in the LFM imaging of the grooves to reduce the tip wear and to minimize the capillary forces which strongly influence the friction forces [40]. The values of the pull-off forces were determined to be *F_pull-off_* = 15 nN inside the microgrooves and *F_pull-off_* = 20 nN on the original Ti-DLN surface, much smaller than the tip load *F_load_* = 200 nN.

The value of the friction force (FF) inside the microgrooves was determined by averaging the FF values over a surface area of 1.5 μm × 37.5 μm size, located along the bottom of the groove. The small width (1.5 μm) of the area allowed us to minimize the effect of the groove slope on the lateral forces being measured. The value of the friction force on the original film surface was determined by averaging of the FF values over two surface areas, each of 10 μm × 37.5 μm size, adjacent to the left and right edges of the LFM image in Figure 11b. For the examined microgrooves (for four beam polarizations θ = 0°, 30°, 60° and 90°) the averaged FF values on the original surface were the same within 10%.

**Figure 12:**
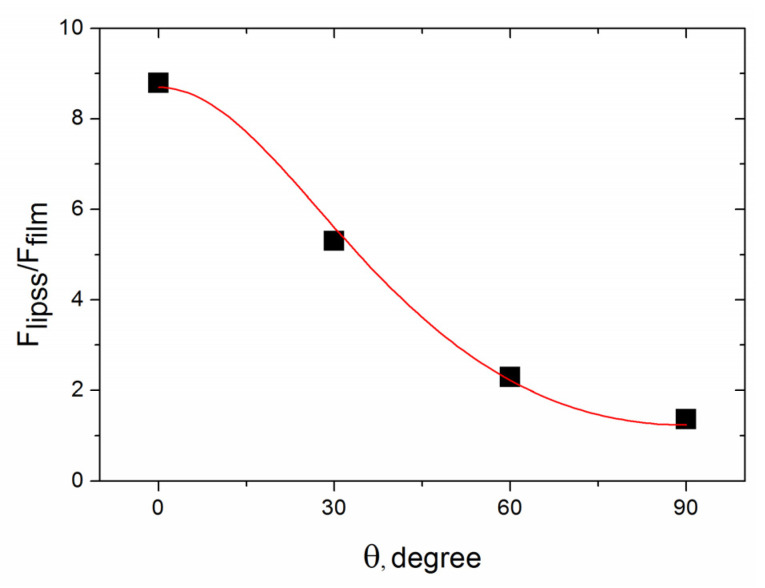
The ratio of the friction forces inside the groove to the friction forces on the original film, *A = F_lipss_/F_film_,* in dependence on the rotation angle of the ripples (corresponding to polarization angle rotation). The red curve is the result of numerical simulations of the *A = F_lipss_/F_film_* for the sinusoidal relief with the maximum slope angle α_sin_ = 60° of the ripples and friction coefficients of the laser-graphitized surface *μ_gr_* = 0.55 and original Ti-DLN film *μ*_0_ = 0.69 (see text for details).

The ratio of the friction forces inside the groove to the friction forces on the original film, *A = F_lipss_/F_film_* is shown in Figure 12 as a function of the polarization angle, identical to the angle of rotation of nanoripples (see Figure 7). It is seen that the FF value on the groove bottom is defined by (i) the presence of the periodical nanostructure and (ii) the angle of rotation of the nanoripples relative to the groove direction (and tip scanning direction). The ratio *A* decreases with the rotation angle. Such behavior is caused by an increase of the average friction force during the tip moving across periodically ascents and descents of the nanoripples, and its dependence on the slope of the ripple surface (along the tip moving line), with the slope decreasing as the rotation angle of the LIPSS is changed from θ = 0° to 90°. It should be noted that accurate calculations of the interrelations between the forces acting on the cantilever tip, and signals registered in the AFM contact-mode—DFL (deflection) and LF (lateral force), are quite complex and require taking into account elastic properties and geometry of both the cantilever and probe as well as the capillary forces [46]. Omitting the details of our estimation of the lateral forces (to be published in our next paper on LFM of laser-nanostructured DLN films), we briefly mention that it was based on the AFM surface profiles of the groove bottom (shown in Figure 6c) and the ripple shape close to a sinusoid with the maximal slope angle of α_sin_ = 60°. Then we performed numerical simulations of the *A = F_lipss_/F_film_* for the bottom relief, assuming it is an ideal periodical array of the sinusoidal ripples with the rotation angle θ relative to the groove bottom and the maximum angle of the relief slope α_sin_ = 60°. For the ‘ideal periodical sinusoidal relief’ the value of *A(θ)*, averaged over a given surface area, is a function of the friction coefficients of the laser-graphitized surface inside the groove (*μ_gr_*) and original film surface (*μ*_0_), and the angles of rotation *θ* and slope *α_sin_(x,y,θ),* under the condition of a linear dependence of the friction force on the tip load (i.e., *F_fr_ = µF_load_*). The best fit of the simulations with the experimental data of *A = F_lipss_/F_film_*, shown in Figure 12 by a red curve, was obtained for the friction coefficients of *μ_gr_* = 0.55 and *μ*_0_ = 0.69.

The obtained friction values correlate with our previous results of the lower friction on the fs-laser-graphitized surface of the superhard carbon films during LFM imaging with a similar diamond-coated tip [47]. In addition, the values of the friction coefficient of a diamond tip on DLC film surface were reported to be *μ* ≈ 0.4 [48], considerably higher than the *μ* values in macroscopic friction measurements. The titanium doping of DLN films leads to higher friction coefficients in macroscopic measurements, increasing from 0.07 (for DLN films) to 0.29 (18%Ti-DLN film) due to the presence of TiC nanocrystals in the DLN matrix [11], with a similar tendency of the higher *μ* values for Ti-DLC films [49]. From this, it follows that the obtained values of the friction coefficients are reasonable. A principle question in the *A(θ)* simulations is concerned with our assumption of *F_fr_ = µF_load_* in the viewpoint of the “friction laws at the nanoscale” [50], which claim that the linear dependence is valid in the case of non-adhesive multi-asperity nanoscale contact. As emphasized above, the measured adhesion force was one order of magnitude smaller than the load force, so it could be neglected in the first approximation. The diamond-coated AFM probe (tip radius of ~100 nm) was produced by deposition of highly doped diamond film during CVD process, i.e., it was a nanocrystalline diamond coating. The nanocrystallinity is thought to provide a multi-asperity diamond contact, especially on a laser-nanostructured surface. Although these questions need to be clarified in further LFM measurements, the proposed approach is of interest in the nanotribology of carbon-based coatings [51] and the potential applications of laser-nanostructured DLC surfaces with variable nanofriction properties.

## 4. Conclusions

The important findings of this work are related to femtosecond visible-laser processing of Ti-DLN films at high pulse frequencies ranging from 100 kHz to 2 MHz, LIPSS fabrication in dependence on the beam polarization rotated with a step of 30° relative to the beam scanning direction, and nanoscale friction properties of the ‘rotating’ LIPSS. Application of the high pulse frequencies is demonstrated to be efficient in fs-laser surface structuring of hard Ti-DLN coatings, but limited by the sub-MHz frequencies due to negative effects of higher surface temperatures (at MHz frequencies) leading to the crack formation and enhanced graphitization of the coatings. Based on the FFT analysis of SEM/AFM images of the LIPSS for different beam polarizations, it is shown that all the LIPSS are oriented perpendicular to the beam polarization direction, and therefore ‘synchronously’ rotated with the electric field vector. It is also found that the LIPSS periods are increased from Λ = 360 ± 5 nm for ripples parallel to the beam scanning direction to Λ = 420 ± 10 nm for ripples formed perpendicular to the laser beam scanning, with the obtained values of Λ = (0.7–0.8)λ > λ/2 corresponding to the formation of low spatial frequency LIPSS at *F > F_th_*. The depth of the formed gratings is in the range of 130–210 nm. The LSFL periods prove to be smaller than the smallest calculated periods of SPP (~470 nm) excited at the air/GC interface, which is supposed to result from the nonnormal beam incidence (changed during ablation processing) with different light polarizations and dielectric permittivity changes of the laser-excited nanocomposite layer.

An interesting approach is proposed to examine nanoscale friction properties of the nanostructured Ti-DLN film using contact-mode AFM techniques and wear-resistant diamond-coated tips. It is based on the analysis of the ratio of the friction forces on the LIPSS-structured surface to the friction forces on the original film, *A = F_lipss_/F_film_*, in dependence on the angle of rotation of nanoripples (identical to the angle of the polarization rotation) relative to the tip scanning direction. The ratio *A* is shown to decrease with the rotation angle θ (for θ = 0° the tip is moving normally to the ripples and for θ = 90°—parallel to the ripples), which is caused by an increase of the average friction force during the tip moving across periodically ascents and descents of the ripples, and its dependence on the slope of the ripple surface along the tip moving line. Numerical simulations of the *A* values for the LIPSS relief, assuming it an ideal periodical array of the sinusoidal ripples with the rotation angle θ, allows also the friction coefficients of the original and laser-graphitized surfaces to be derived from the best fit with the experimental values of A.

The obtained results demonstrate that femtosecond laser ablation processing is an effective technique to fabricate LIPSS of different orientations on the Ti-DLN coatings and control their friction properties at the nanoscale.

## Data Availability

Not applicable.
